# The mitochondrial transporter SLC25A43 is frequently deleted and may influence cell proliferation in HER2-positive breast tumors

**DOI:** 10.1186/1471-2407-12-350

**Published:** 2012-08-10

**Authors:** Elisabet Tina, Breezy Malakkaran Lindqvist, Marike Gabrielson, Zelmina Lubovac, Pia Wegman, Sten Wingren

**Affiliations:** 1 Clinical Research Centre, Örebro University Hospital, SE-70185, Örebro, Sweden; 2 School of Health and Medical Sciences, Örebro University, SE-70182, Örebro, Sweden; 3 School of Life Sciences, University of Skövde, SE-54128, Skövde, Sweden

**Keywords:** Breast cancer, HER2, SLC25A43, S-phase

## Abstract

**Background:**

Overexpression of the human epidermal growth factor receptor (HER) 2 is associated with poor prognosis and shortened survival in breast cancer patients. HER2 is a potent activator of several signaling pathways that support cell survival, proliferation and metabolism. In HER2-positive breast cancer there are most likely unexplored proteins that act directly or indirectly downstream of well established pathways and take part in tumor development and treatment response.

**Methods:**

In order to identify novel copy number variations (CNVs) in HER2-positive breast cancer whole-genome single nucleotide polymorphism (SNP) arrays were used. A PCR-based loss of heterozygosis (LOH) assay was conducted to verify presence of deletion in HER2-positive breast cancer cases but also in HER2 negative breast cancers, cervical cancers and lung cancers. Screening for mutations was performed using single-strand conformation polymorphism (SSCP) followed by PCR sequencing. Protein expression was evaluated with immunohistochemistry (IHC).

**Results:**

A common deletion at chromosome Xq24 was found in 80% of the cases. This locus harbors the gene solute carrier (SLC) family 25A member 43 (SLC25A43) encoding for a mitochondrial transport protein. The LOH assay revealed presence of *SLC25A43* deletion in HER2-positive (48%), HER2-negative (9%), cervical (42%) and lung (67%) cancers. HER2-positive tumors with negative or low SLC25A43 protein expression had significantly lower S-phase fraction compared to tumors with medium or high expression (*P* = 0.024).

**Conclusions:**

We have found deletion in the *SLC25A43* gene to be a common event in HER2-positive breast cancer as well as in other cancers. In addition, the SLC25A43 protein expression was shown to be related to S-phase fraction in HER2-positive breast cancer. Our results indicate a possible role of SLC25A43 in HER2-positive breast cancer and support the hypothesis of altered mitochondrial function in cancer.

## Background

In approximately 20% of primary breast cancers, the tumor cells have acquired a capacity to increase their survival and proliferation by overexpressing the human epidermal growth factor receptor (HER) 2 [1,2]. This overexpression of the HER2 protein is in the majority of cases caused by gene amplification [3]. Patients diagnosed with a HER2-positive tumor have in general a higher risk for relapse, shorter time to disease progression, and poorer survival [1,4,5]. For over a decade, the recombinant humanized monoclonal antibody Trastuzumab has been used in clinic for treatment of HER2-positve breast cancer which has improved the outcome for these patients [6]. However, acquired resistance to Trastuzumab is common among patients who initially respond, even when combined with chemotherapy [7-9]. For patients with tumor progression after treatment with Trastuzumab, lapatinib provides a new treatment option. Lapatinib is a small molecule that competitively binds to HER2 but also to the family member HER1, and thereby decreases activation of downstream signals [6].

Several signaling pathways associated with cell proliferation, cell growth, differentiation, survival and metabolism are activated by the HER-family [2]. The Phoshatidylinositol-3-kinase (PI3K) pathway has been identified to have a significant role in HER2-positive breast cancer as well as in the development and progression of other cancers. Studies have shown that the PI3K pathway is frequently perturbed as a result of genetic alterations such as activating mutations in the *PIK3CA* gene [10], or mutation or deletion of the negative regulator phosphatase and tensin homologue (PTEN) [11,12]. These genetic alterations can lead to constitutive activation of the PI3K pathway and thereby contribute to treatment failure [13,14]. The HER2 network contributes to a plethora of cellular functions and there are most likely unexplored proteins acting directly or indirectly of downstream pathways affecting tumor development and treatment response.

The aim of the study was to identify novel copy number variations (CNVs), underlying development and progression of HER2-positive breast cancer, using whole-genome single nucleotide polymorphism arrays. The result revealed a common deletion of the gene encoding for the inner mitochondrial membrane transporter, solute carrier (SLC) family 25A member 43 (SLC25A43). Furthermore, the SLC25A43 protein was found to have a possible role in cell proliferation.

## Methods

### Patient material

Tumor samples from 85 patients with HER2-positive breast cancer diagnosed between 1993 and 2008 were included in the study. Overexpression and/or gene amplification of HER2 were confirmed using immunohistochemistry (HercepTest™ (Dako, Glostrup, Denmark)) and fluorescence *in situ* hybridization (PathVysion HER-2 DNA Probe Kit (Abbott, Illinois, USA)). Both were performed according to manufacturer’s protocols. Retrospectively collected patient characteristics for the HER2-positive breast cancer cohort are shown in Table 1. The S-phase fraction was determined using a flow cytometry assay based on Vindelöv *et al*[15] and hormone receptor status was evaluated using immunohistochemistry. HER2 status, S-phase and hormone receptors were all assessed at time of diagnosis.

**Table 1:** Patient characteristics and the SLC25A43 expression in the HER2-positive breast cancer cohort

**Parameters**	**No. of cases total in the cohort (%)**	**Mean values of total cases in the cohort**	**SLC25A43 expression**		***P-*value**
			**Negative/Low**	**Medium/High**	
All	85		26	45	
Patient's age					
≤50 years	22 (26)				
>50 years	62 (73)				
Mean age (years)		58.5	62	58	0.269
ND	1 (1)				
Tumour size					
≤2 cm	33 (39)				
>2 cm	50 (59)				
Mean size (mm)		28	25	30	0.13
ND	2 (2)				
S-phase					
Include cases	70 (82)				
Mean S-phase (%)		10.7	9.4	12.6	0.024
ND	15 (18)				
LN metastasis					
Negative	27 (32)		8	15	0.859
Positive	55 (65)		17	29	
ND	3 (4)				
ER status					
Negative	38 (45)		12	20	0.951
Positive	41 (48)		13	21	
ND	6 (7)				
PgR status					
Negative	45 (53)		16	24	0.659
Positive	33 (39)		9	17	
ND	7 (8)				

*LN*, lymph node; *ER*, oestrogen receptor; *PgR*, progesterone receptor; *ND*, not determined.

To further analyze presence of loss of heterozygosis in *SLC25A43* in other malignancies, we included cohorts of HER2-negative breast cancers, cervical cancer and lung cancer. The cohort of HER2-negative, estrogen receptor (ER) positive breast cancers consisted of samples from 52 patients with a median age of 64 years. Cervical tumor samples were obtained from 65 patients (median age of 69 years). Finally, the cohort of lung cancers included 18 female patients (median age 63 years) diagnosed with adenocarcinoma.

A study layout of the patient material is shown in Table 2.

**Table 2:** Study layout of patient material

**Patient cohorts**	**Number of patients**
HER2-positive BC	85
SNP array	25
LOH	85
Mutation analysis	29
IHC	71
HER2-negative BC^a^	52
Cervical cancer^a^	65
Lung cancer (♀)^a^	18

^a^, patients used for *LOH* analysis; *BC*, breast cancer; *SNP*, single nucleotide polymorphism; *LOH*, loss of heterozygosity; *IHC*, immunohistochemistry.

The research ethical committee at the Faculty of Health Science, Linköping, Sweden, approved this study with no request of an informed consent.

### Isolation of DNA

DNA was isolated from approximately 10 mg of fresh frozen tissue and Buffy coat samples from female blood donor, using QIAamp® DNA Mini Kit or EZI DNA,QIAGEN® Tissue kit (both from QIAGEN Inc., Valencia, CA, USA) followed by purification using BioRobot EZI workstation (QIAGEN Inc.). DNA concentrations were quantified using NanoDrop® ND-1000 UV–vis Spectrophotometer (NanoDrop Technologies, Wilmington, DE, USA).

### Whole-genome assay

Whole-genome screening of CNVs was performed using Gene Chip® Mapping 250 K Assay Kit (Affymetrix, Inc Santa Clara, CA USA) according to manufacturer’s protocol. Briefly, 250 ng of DNA was digested with *NspI* restriction enzyme. The DNA fragments were then ligated with an adaptor and PCR amplified with general primers. The PCR product was purified, quantified and cleaved to shorter fragments, followed by labeling. Thereafter, the sample was hybridized to the array and then stained with a streptavidin-biotin solution. A Gene Chip Scanner 3000 7 G (Affymetrix) was used to scan the chip. To generate a segment report, which is a table where each row corresponds to a segment with copy number change that deviates from the expected normal, Genotyping Consol 3.1 (Affymetrix) was used. Array data from forty-six female individuals, available from the International Hap Map project, were used as controls for known CNV regions. To generate the segment report, three different criteria were applied by setting different values of the parameters provided by the application. One of the parameters is defined as the number of SNPs within the segment. By setting the value of this parameter to five, we introduce constraints to only identify segments with a minimum of five SNPs within the segment. The other parameter defined the size of the reported segment to a minimum size of 100 kbp. Finally, a parameter for CNV overlap was set to report all segments with up to 50% overlap with known CNV regions. For each segment, there is an indication of loss or gain, depending on whether the copy number change correspond to a decrease or increase from what is normally expected. Further bioinformatics analysis consisted of selecting the most frequent aberrations, present in a minimum of 10 tumors, and applying clustering tools to find common patterns of genetic changes among different patients [16].

### Loss of heterozygosis assay

Loss of heterozygosis (LOH) analysis of the *SLC25A43* gene was carried out by a PCR- restriction fragment length polymorphism (PCR-RFLP) based method. A polymorphic site, common among the European/Scandinavian population (SNP ID: rs217978), between exon 1 and 2 in the *SLC25A43* gene was chosen for the analysis. PCR amplification was performed in a final reaction volume of 20 μl containing 25-50 ng of DNA, 1x TopTaq PCR buffer (1.5 mM MgCl_2_ (Qiagen) 0.2 mM of each dNTP, 0.025 U TopTaq DNA polymerase (Qiagen), 1x Q-solution™ (Qiagen) and 1 μM of primers flanking the SNP (5’-GCATTGGGAGAATGAGGAGT-3’, HEX5’-CTGTTGTGCTGCTTGCCTTC-3’ respectively). Amplification was carried out using the following conditions: initial denaturation (94°C for 3 min), followed by 35 cycles of denaturation (94°C for 30 sec), annealing (60°C for 30 sec) and extension (72°C for 60 sec, 5) ending with a final extension at 72°C for 10 min. The 101 bp PCR product was then cleaved at the polymorphic site using *Acc1* (New England BioLabs Inc., Ipswich, MA, US) at 37°C for 8 hours followed by heat inactivation at 80°C for 20 minutes. The cleaved products were resolved by 3% agarose (w/v) gel electrophoresis and all samples heterozygous for rs.217978 were selected for capillary electrophoresis in an ABI Prism® 3130 Genetic Analyzer (Applied Biosystems, CA, USA). GeneMapper v 4.0 software was used for the analysis of the area under the peak which represents the amount of each allele. For each sample, an allelic ratio (AR) was calculated by dividing the peak area of allele 1 by the peak area of allele 2. To determine a reference interval (mean allelic ratio ± 1.96SD), 29 heterozygous female blood donors were used and tumor samples with an AR within the reference interval were defined to have retention of heterozygosis (ROH) while samples outside the range were considered to have LOH.

### Mutation analysis

All exons of the *SLC25A43* gene were screened in HER2-positve breast cancer samples using single-strand conformation polymorphism (SSCP). The primary PCR was performed in a total volume of 20 μl PCR containing 25-50 ng DNA, 1x TopTaq PCR buffer (1.5 mM MgCl_2,_ 0.2 mM of each dNTP) (Qiagen), 0.025U TopTaq DNA polymerase (Qiagen), 1x Q-solution™ (Qiagen) and 1 μM of each primer (Additional file 1: Table S1). Amplification was carried out using the following conditions: initial denaturation (94°C for 3 min), followed by 30 cycles of denaturation (94°C for 30 sec), annealing (59 or 60°C for 30 sec) and extension (72°C for 60 sec) ending with a final extension (72°C for 10 min). These amplicons were then radiolabeled for single-strand conformation polymorphism (SSCP) in a secondary PCR, where 2 μl of the primary PCR product was amplified for 12 cycles in the presence of deoxyadenosine 5’-triphosphate, α-^33^P (Perkin Elmer, Boston, MA, USA). Fragments were run both on a non-denaturing 6% polyacrylamide gel at 6 W (3000 V, 300 mA) for 16-18 hours and on a MDE™ gel (Mutation Detection Enhancement, BMA products, Rockland, ME) for 8 hours at room temperature. The gel was dried and auto exposed to X-ray film (35 x 45 cm, Kodak BMS-1) in an exposure cassette using Trans Screen LE intensifier at -80°C for 1-2 days. The DNA fragments with mobility shifts were excised and preamplifier for sequencing by PCR. Cycle sequencing was performed using a BigDye® Terminator kit (Applied Biosystems, CA, USA) on 1 μl of clean PCR product (PCR-M ™ clean up system, VIOGENE, Sunnyvale, CA, USA) in a final volume of 10 μl containing 0.5x BigDye Terminator v1.1 Ready Reaction Mix, 0.5x BigDye Sequencing buffer, 0.16 μM of forward and reverse primers in a 2720 thermal cycler (Applied Biosystems, CA, USA) using following conditions; Initial denaturation (96°C for 60 sec), followed by 25 cycles of denaturation (96°C for 10 sec), annealing (50°C for 5 sec) and extension (60°C for 4 sec). The extension products were purified using an ethanol (95%) - Sodium acetate (3 M, pH 4.6) precipitation technique followed by capillary electrophoresis in an ABI Prism® 3130 Genetic Analyzer (Applied Biosystems CA, USA). A partial sequence of pGEM-3Zf (+) was used as control sequence. The results were analyzed using Sequencing Analysis v5.2 (CA, USA) and ChromasPro v1.34 developed by Technelysium Pty, Ltd.

### Immunohistochemistry

**Figure 1:**
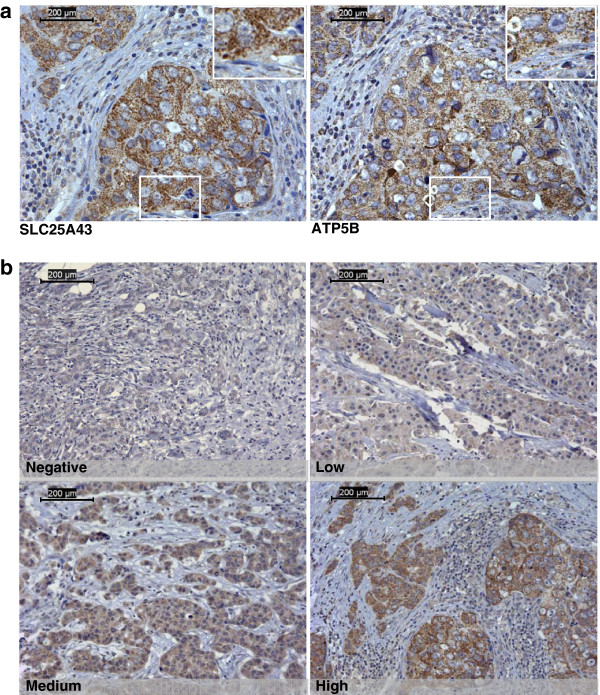
**Protein expression of SLC24A43 in HER2-positive tumours on TMA.** Staining of SLC25A43 is localised to the cytoplasm of the cells. Comparison of staining localisation and pattern between SLC25A43 and the mitochondrial inner membrane protein ATP5B in breast tumours (x40 Objective) (**a**). The four different levels of SLC24A43 expression in HER2-positive tumours used for scoring (x20 Objective) (**b**). Scale bar is showing 200 μm.

Formalin-fixed tissue micro array (TMA) sections were deparaffinised, rehydrated and washed. Endogenous peroxidase were blocked using 3% hydrogen peroxide, followed by heat-induced antigen retrieval in 1 mM EDTA buffer pH 8.0 for 20 minutes. Slides were incubated with primary antibody rabbit polyclonal anti-SLC25A43 (1:500) (raised against the C-terminal part of the protein (Agrisera, Vännäs, Sweden)) for 20 minutes at room temperature. Slides were washed and subsequently incubated with horseradish peroxidase-conjugated secondary antibody (Dako) for 20 minutes at room temperature. Bound antibodies were visualized using diaminobenzidine (DAB) chromogen (Dako) and counterstained with haematoxylin. The specificity of the antibody was tested by western blot with the synthetic peptide used as the immunogen (data not shown). The mitochondrial inner membrane protein ATP5B was used as comparison for localization of immunostaining (Figure 1a). For scoring of the SLC25A43 expression, an intensity scale was applied using the following criteria: 0: negative expression, 1+: low expression, 2+: medium expression and 3+: high expression (Figure 1b). The slides were scored by two independent observers without knowledge about clinical and pathological data.

### Statistical analysis

To evaluate the association between LOH, IHC and ER/PgR-status or positive lymph nodes, *χ*^2^ test were performed. Differences in age, tumor size or S-phase between LOH/ROH and IHC scoring were tested by Student’s *T*-test. Statistical significance was set at *P* < 0.05. SPSS 17.0 statistical software for Windows (SPSS Inc., Chicago, IL, USA) was used for all tests.

## Results

### Deletion at Xq24 in HER2-positive breast cancer

**Figure 2:**
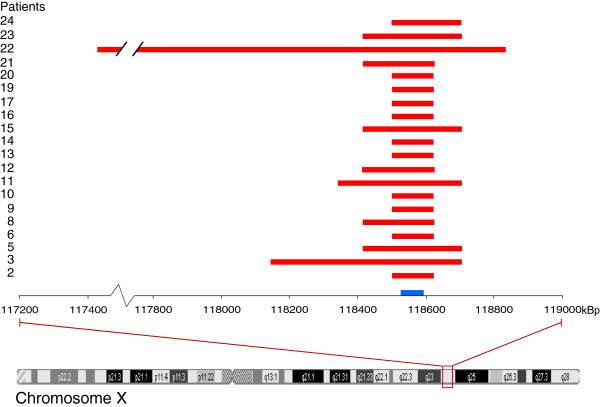
**Deletions at Xq24 in HER2-positive cancer.** Tumour samples were analysed using whole-genome array to identify CNVs. Start and end points for the deletion found in 20 of 25 cases at Xq24. The position of *SLC25A43* is marked blue on the X-axis.

In our search for novel CNVs in HER2-positive breast cancers, 25 tumors were screened using a whole-genome array. To identify the most common CNVs, we only proceeded with events that were found in at least ten of the tumors. Using this approach we obtained CNV in 40 different loci whereof 16 were amplifications and 24 deletions. Most of the loci with amplification were seen in chromosome 1q but there were also amplifications found in specific loci in 3q, 8q, 12q, 17q and in both arms of the X chromosome. Deletions were mostly presented at 11q, 17p and Xq. In 80% of the 25 HER2-positive breast tumors deletion at Xq24 was observed, covering both previously known genes as well as novel genes in relation to cancer (Additional file 1: Table S2 and S3). The deletion pattern at Xq24 is shown in Figure 2. In all these tumors, the deletion at Xq24 encompassed the two genes *SLC25A43* and *SLC25A5*, which both encodes for inner mitochondrial membrane transporters of the SLC25 family [17-19]. SLC25A5 translocates adenosine diphosphate (ADP) from the mitochondrial matrix into the cytoplasm and partially share its function with the homolog SLC25A6 (91% amino acid similarity) [17,20]. SLC25A43 is suggested to be an importer although its preferred substrate is currently not known [21]. The common deletion found covering *SLC25A43* indicates a possible role of this gene in HER2-positve breast cancer. Since SLC25A43 has not been previously studied in relation to cancer we decided to further investigate this finding.

### Common loss of heterozygosity in *SLC25A43*

We continued to verify the *SLC25A43* deletion identified by whole-genome array, using a PCR-based LOH assay in 85 HER2-positive breast cancer cases. Cases that showed a heterozygous pattern after enzymatic cleavage were used for further LOH analyses (Table 3). Based on the reference interval (0.39 – 1.25), 48% of the heterozygous cases showed allelic ratios outside the range and were defined as having LOH. When testing for associations between LOH and tumor characteristics no statistical significance was found. Although, age at diagnosis was significantly higher in the LOH group when compared with the ROH group, with the mean age of 67 and 49 years, respectively (*P* = 0.0002). In addition, to evaluate if the deletion was a general event in cancer among women we added tumor samples from HER2-negative breast cancer, cervical cancer and lung cancer in the LOH analysis. The three cohorts showed frequencies of 9%, 42% and 67%, respectively (Table 3).

**Table 3:** Loss of heterozygosity in different tumours

**Type of cancer**	**Heterozygous patients (%)**	**Patients with LOH (%)**
HER2-positive	33/85 (39)	16/33 (48)
HER2-negative	22/52 (42)	2/22 (9)
Cervical cancer	26/65 (40)	11/26 (42)
Lung cancer (♀)	6/18 (33)	4/6 (67)

*LOH*, loss of heterozygosity.

The high frequency of LOH found in *SLC25A43* indicated a possible role as a tumor suppressor gene. To investigate presence of a possible second genetic alteration in the *SLC25A43* gene, ensuring complete inactivation of a tumor suppressor gene [22], we screened 29 HER2-positive breast tumors for mutations. However, we could not detect any mutation.

### SLC25A43 expression varies in HER2-positive tumors

**Figure 3:**
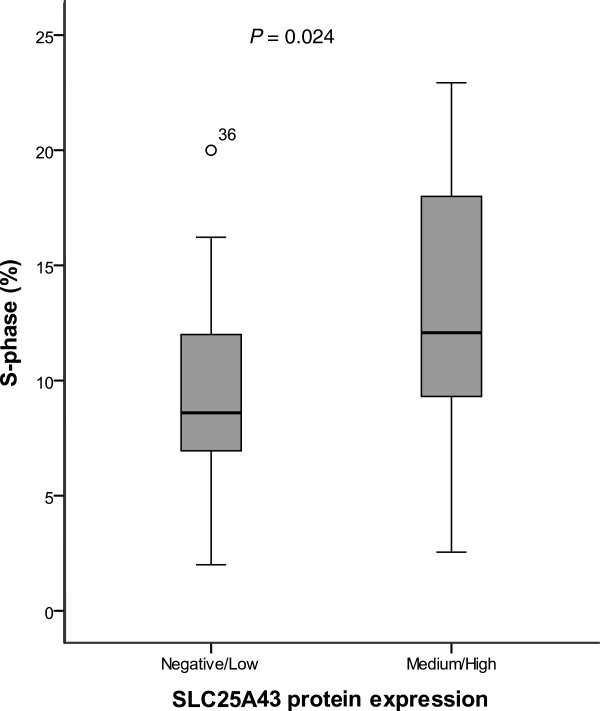
**Boxplot of S-phase distribution and SLC24A43 protein expression.** S-phase fraction in tumours with negative/low protein expression (n = 26) and medium/high expression (n = 45) of SLC25A43.

Out of the 85 HER2-positive tumors, 71 cases were available for further analysis using TMA in order to evaluate protein expression of SLC25A43. The immunostained tumor cells demonstrated a homogeneous expression of SLC25A43 within the tumor but the expression varied widely, spanning from non-existent to very high expression, between tumors (Figure 1b). Four tumors were negative and 22 tumors had low expression, while 28 and 17 tumors showed medium and high expression, respectively. In the statistical analysis we combined cases with negative and low expression (37%) to compare with a combination of the cases showing medium or high expression (63%). There was no significant association between SLC25A43 expression and LOH, which could in part be explained by the few number of cases with LOH in the *SLC25A43* gene (n = 16) that were available for comparison. SLC25A43 expression was not associated with ER status, lymph node metastasis or age at diagnosis (Table 1). However, cases with negative or low expression of SLC25A43 did show a significantly lower Synthesis (S)-phase fraction (mean S-phase 9.4%) compared to cases with medium or high expression (mean S-phase 12.6%) (*P* = 0.024) (Table 1, Figure 3).

## Discussion

In this study, a common deletion of the *SLC25A43* gene, located at Xq24, was revealed in HER2-positive breast cancer using whole-genome array. This novel finding indicates a possible role of SLC25A43 in HER2-positive breast cancer through an altered mitochondrial function.

CNVs have previously been reported at Xq in HER2-positive breast cancer [23,24], supporting our finding. Deletions in X chromosome are also suggested to be associated with tumor progression, metastasis and poor prognosis in breast cancer [25]. We further confirmed deletion in the *SLC25A43* gene in a larger HER2-positive cohort using a PCR-based LOH assay. Deletion in *SLC25A43* was also found to be present in other types of cancer in female patients, suggesting it to be a putative tumor suppressor gene. However, there were no mutations detected within the *SLC25A43* gene in our cohort. Another possible mechanism for transcriptional silencing is increased methylation [26,27], and recent findings show higher methylation in the CpG island of the *SLC25A43* gene in breast cancer in the absence of gene deletion [28]. Higher CpG island methylation was also associated with lower age at diagnosis in HER2-positive breast cancer [28] while deletion of *SLC25A43* was found in tumors from patients with higher age at diagnosis. In females one of the two X chromosomes is randomly inactivated by DNA methylation [29], thus the need of two hits for complete inactivation of *SLC25A43* depends on whether the deletion is localized on the active or inactive X chromosome. The limitation with the two methods used in the present study to determine occurrence of *SLC25A43* deletion is that they do not discriminate between the active or inactive X chromosome.

When evaluating the protein expression with immunohistochemistry, results showed that SLC25A43 expression varied between negative to high among HER2-positive breast cancers and was not associated with ER status, lymph node metastasis or age at diagnosis. A possible explanation for the lack of association between LOH-status and protein expression could be the low number of cases available for comparison. It would therefore be of great interest to expand the cohort including cases with deletion in the SLC25A43 gene in the future. However, in our study the S-phase fraction was shown to be significantly lower in cases with negative or low SLC25A43 expression compared with medium or high expression of SLC25A43. It is currently not possible to explain the relation between SLC25A43 expression and S-phase fraction. Mitra *et al* have shown that the mitochondria also could have a role in cell cycle regulation [30] but the connection between SLC25A43 and cell proliferation has to be further elucidated.

The closest relative of SLC25A43 is the Coenzyme A (CoA) transporter SLC25A16, sharing 29% amino acid sequence similarity [18]. The next closest relative, SLC25A42, has been shown to transport both CoA and ADP [31]. Even though the function of SLC25A43 remains unexplored, alterations in the expression is likely to affect mitochondrial function. Mitochondrial dysfunction in cancer cells was first described by Otto Warburg in the 1920s where he demonstrated an increased glycolysis in the presence of oxygen (aerobic glycolysis) [32]. Today, it is well established that mitochondrial dysfunction have an important role in tumor development and progression. Hanahan and Weinberg proposed that one of the hallmark capabilities in cancer cells is their enhanced resistance to mitochondrial apoptosis [33]. Another hallmark is the sustained chronic proliferation found in cancer cells as a result of evading the strictly controlled production and release of growth factors as well as dysregulation of receptors and downstream signals [33].

Our findings of deletion in the *SLC25A43* gene together with varied SLC25A43 protein expression in HER2-positive breast cancers supports the role of altered mitochondrial function as a hallmark of cancer.

## Conclusions

To the best of our knowledge, this is the first report to demonstrate a common deletion in *SLC25A43* in HER2-positive breast cancer as well as in other cancers, suggesting that it might be an important genetic event. The SLC25A43 protein expression was found to be related to S-phase fraction indicating a possible role of this mitochondrial protein in cell proliferation.

## Competing interests

The authors declare that they have no competing interests.

## Authors’ contributions

All authors contributed meaningfully to this manuscript, in particular: ET, BML and MG performed laboratory work and drafted the manuscript; ZL conducted the data analysis of whole-genome data; MG carried out statistical analysis; PW supported the manuscript writing; SW proposed and designed this study. All authors have read and approved the final manuscript.

## Pre-publication history

The pre-publication history for this paper can be accessed here:

http://www.biomedcentral.com/1471-2407/12/350/prepub

## Supplementary Material

Additional file 1 **Table S1. Primer sequences used for PCR in the mutation analysis of the*SLC25A43*gene.** A table showing primer sequences used for the mutation analysis of *SLC25A43* gene. Table S2. Genomic position and size of deletion at Xq24 in HER2-positive breast cancer cases as revealed by the whole-genome array (Based on *Homo sapiens* build 37.3). A table showing the extend of deletion at Xq24 in HER2-positive breast cancers. Table 3 List of genes deleted at Xq24 in HER2-positive breast cancer as revealed by the whole-genome array. A table showing the number patients that have deletion at Xq24, the genes affected and references for those that are previously implicated in cancer. Click here for file
