# Supplementary material for: The mitochondrial transporter SLC25A43 is frequently deleted and may influence cell proliferation in HER2-positive breast tumors
Source: BMC Cancer. 2012 Aug 10;12:350. doi: 10.1186/1471-2407-12-350 (PMC3462703; doi:10.1186/1471-2407-12-350)
Supplement: Additional file 1 — Table S1. Primer sequences used for PCR in the mutation analysis of theSLC25A43gene. A table showing primer sequences used for the mutation analysis of SLC25A43 gene. Table S2. Genomic position and size of deletion at Xq24 in HER2-positive breast cancer cases as revealed by the whole-genome array (Based on Homo sapiens build 37.3). A table showing the extend of deletion at Xq24 in HER2-positive breast cancers. Table 3 List of genes deleted at Xq24 in HER2-positive breast cancer as revealed by the whole-genome array. A table showing the number patients that have deletion at Xq24, the genes affected and references for those that are previously implicated in cancer. [file 1471-2407-12-350-S1.pdf]

ADDITIONAL FILE

The mitochondrial transporter SLC25A43 is frequently deleted and may influence cell proliferation in HER2-positive breast tumours

Elisabet Tina[1,2,4,*], Breezy Malakkaran Lindqvist[2,4], Marike Gabrielson[2,4], Zelmina Lubovac[3], Pia Wegman[2] and Sten Wingren[2]

Additional file Table 1 Primer sequences used for PCR in the mutation analysis of the *SLC25A43* gene.

**Exon**	**Forward (5´→3´)**	**Reverse (5´→3´)**
1A	GCCAGGCTAGGGCGTAGA	CTTTGGCCGCCTGTCAGT
1B	ACTGACAGGCGGCCAAAG	GGCTCTTACTTGCGGTAGGC
2	GCCTTTCCCCCTATTTTCTC	GGACAAGGTGAAAAGGTCCA
3	CTTCTCCCTGGCCTTGTTT	CCCCACTCTGATAACTCCTCA
4	TGGGAGCATTCACAGGAAGT	GGCACCATTCTTACCCTCAA
5	TCCCAGCTTCTGTTTCCTGT	GAGGCTGGTGGCTCTTCTC
6	TTGATTTTCATTTTTGGTTTTAACA	TGAGGCTACATTTATCTGTGATTG

Additional file Table 2 Genomic positions and size of deletion at Xq24 in HER2-positive breast cancer cases as revealed by the whole genome array (Based on *Homo sapiens* build 37.3).

**Patient ID**	**Start**	**Size loss (kBp)**
2	118504.82	110.559
3	118148.23	551.091
5	118419.195	280.126
6	118504.82	110.559
8	118419.195	196.184
9	118504.82	110.559
10	118504.82	110.559
11	118348.041	351.28
12	118419.195	196.184
13	118504.82	110.559
14	118504.82	110.559
15	118419.195	280.126
16	118504.82	110.559
17	118504.82	110.559
19	118504.82	110.559
20	118504.82	110.559
21	118419.195	196.184
22	117442.441	1388.026
23	118419.195	280.126
24	118504.82	194.501

Additional file Table 3 List of genes deleted at Xq24 in HER2-positive breast cancer as revealed by the whole genome array.

No of Patients	Gene in the deleted locus	References*
20	*SLC25A43*	-
20	***SLC25A5***	[1, 2]
5	*CXorf56*	-
3	***PGRMC1***	[3, 4]
2	***KIAA1210***	[5]
2	*LONRF3*	-
1	***ZCCHC12***	[6]
1	***IL13RA1***	[7, 8]
1	***DOCK11***	[9]
1	*WDR44*	-
1	*UBE2A*	-
1	***NKRF***	[10]
1	*MIR766*	-
1	***SEPT6***	[11, 12]

*References for the genes that are previously implicated in **breast cancer** and **other cancers**

Additional file References

1. Jang JY, Choi Y, Jeon YK, Kim CW: **Suppression of adenine nucleotide translocase-2 by vector-based siRNA in human breast cancer cells induces apoptosis and inhibits tumor growth in vitro and in vivo**. *Breast Cancer Res* 2008, **10**(1):R11.
2. Chevrollier A, Loiseau D, Reynier P, Stepien G: **Adenine nucleotide translocase 2 is a key mitochondrial protein in cancer metabolism**. *Biochim Biophys Acta* 2010, **1807**(6):562-567.
3. Neubauer H, Yang Y, Seeger H, Fehm T, Cahill MA, Tong X, Ruan X, Mueck AO: **The presence of a membrane-bound progesterone receptor sensitizes the estradiol-induced effect on the proliferation of human breast cancer cells.** *Menopause* 2011, **18**(8):845-850.
4. Craven RJ: **PGRMC1: a new biomarker for the estrogen receptor in breast cancer.** *Breast Cancer Res* 2008, **10**(6):113.
5. Vanaja DK, Ballman KV, Morlan BW, Cheville JC, Neumann RM, Lieber MM, Tindall DJ, Young CY: **PDLIM4 repression by hypermethylation as a potential biomarker for prostate cancer.** *Clin Cancer Res* 2006, **12**(4):1128-1136.
6. Li QL, Chen FJ, Lai R, Guo ZM, Luo R, Yang AK: **ZCCHC12, a potential molecular marker of papillary thyroid carcinoma: a preliminary study.** *Med Oncol* 2011.
7. Serão NV, Delfino KR, Southey BR, Beever JE, Rodriguez-Zas SL: **Cell cycle and aging, morphogenesis, and response to stimuli genes are individualized biomarkers of glioblastoma progression and survival.** *BMC Med Genomics* 2011, **4**:49.
8. Staege MS, Banning-Eichenseer U, Weissflog G, Volkmer I, Burdach S, Richter G, Mauz-Körholz C, Föll J, Körholz D: **Gene expression profiles of Hodgkin's lymphoma cell lines with different sensitivity to cytotoxic drugs.** *Exp Hematol* 2008, **36**(7):886-896.
9. Almstrup K, Leffers H, Lothe RA, Skakkebaek NE, Sonne SB, Nielsen JE, Rajpert-De Meyts E, Skotheim RI: **Improved gene expression signature of testicular carcinoma in situ.** *Int J Androl* 2007, **30**(4):292-302; discussion 303.
10. Lu Z, Li Y, Takwi A, Li B, Zhang J, Conklin DJ, Young KH, Martin R: **miR-301a as an NF-κB activator in pancreatic cancer cells.** *EMBO J* 2011, **30**(1):57-67.
11. Cerveira N, Santos J, Teixeira MR: **Structural and expression changes of septins in myeloid neoplasia.** *Crit Rev Oncog* 2009, **15**(1-2):91-115.
12. Cerveira N, Bizarro S, Teixeira MR: **MLL-SEPTIN gene fusions in hematological malignancies.** *Biol Chem* 2011, **392**(8-9):713-724.
